# The Plasma Membrane H+-ATPase Promoter Driving the Expression of *FADX* Enables Highly Efficient Production of Punicic Acid in *Rhodotorula toruloides* Cultivated on Glucose and Crude Glycerol

**DOI:** 10.3390/jof10090649

**Published:** 2024-09-13

**Authors:** Daniela Krajciova, Roman Holic

**Affiliations:** Institute of Animal Biochemistry and Genetics, Centre of Biosciences, Slovak Academy of Sciences, Dubravska Cesta 9, 84005 Bratislava, Slovakia; daniela.krajciova@savba.sk

**Keywords:** synthetic biology, metabolic engineering, *Rhodotorula toruloides*, conjugated linolenic acid (CLNA), FADX, single-cell oil, lipid, crude glycerol

## Abstract

Punicic acid (PuA) is a conjugated fatty acid with a wide range of nutraceutical properties naturally present in pomegranate seed oil. To meet the rising demand for pomegranate seed oil, a single-cell oil enriched in PuA provides a sustainable biomass-derived alternative. This study describes the production of a PuA-enriched single-cell oil through the engineering of the red yeast *Rhodotorula toruloides* grown in glucose and a low-cost substrate, crude glycerol. The gene for *Punica granatum* fatty acid conjugase, *PgFADX*, was randomly integrated into the genome of *R. toruloides* without disrupting the carotenoid synthesis. In shake flask studies, the effects of three promoters (P*_PGI1_*, P*_NAR1_*, and P*_PMA1_*) on PuA production were evaluated. PuA titers of 105.77 mg/L and 72.81 mg/L were obtained from engineered cells expressing *PgFADX* from the P*_PMA1_* promoter cultivated for 72 h in glucose and for 168 h in crude glycerol, respectively. Furthermore, the detailed lipid analysis revealed a high enrichment PuA in the triacylglycerol lipid structures, even without substantial modifications to the metabolic pathways. This report demonstrates the high potential of *R. toruloides* in the upcycling of a low-cost substrate, crude glycerol, into a value-added product such as PuA. The findings support the feasibility of using engineered *R. toruloides* for sustainable production of PuA-enriched single-cell oil.

## 1. Introduction

Yeasts are commonly utilized in various biotechnological applications, including the production of biofuels, specialty chemicals, polymers, biomaterials, pharmaceuticals, enzymes, and recombinant proteins. They efficiently metabolize sugars as well as other carbon sources and, thanks to modern methods of genetic engineering, yeast-based production systems are sustainable and renewable, and have minimal environmental impact [1].

The oleaginous yeast *Rhodotorula* (*Rhodosporidium*) *toruloides* is capable of high-density growth while simultaneously producing a high titer of lipids [2]. It is also known as “the red yeast” due to its production of carotenoids, which results in its characteristic red coloration. Carotenoids, including beta-carotene, torulene, and torularhodin, are valuable for their antioxidant properties and their applications in the food, pharmaceutical, and cosmetic industries [3,4]. In addition, *R. toruloides* naturally utilizes various carbon sources for growth, including glucose, xylose, cellobiose, glycerol, acetic acid, and cellulose biomass hydrolysates [5,6,7,8], and exhibits halotolerance [9]. Like other yeasts, significant lipid accumulation occurs under nitrogen-limited conditions [10]. *R. toruloides* has immense potential for the industrial production of value-added lipids. Every year, advances in molecular tools simplify genetic manipulations in this yeast, overcoming challenges in its utilization in both scientific research and industry [11].

Punicic acid (PuA, C18:3Delta9*cis*,11*trans*,13*cis*) with three conjugated double bonds is an isomer of alpha-linolenic acid (C18:3Delta9*cis*,12*cis*,15*cis*), and it constitutes approximately 60–80% of pomegranate seed oil (PSO) [12]. Oils enriched with conjugated fatty acids are valuable for their nutritional value and industrial applications. Currently, significant attention is focused on the anticancer effects of PSO. It inhibits oxidation and prostaglandin synthesis, reduces the incidence of breast, prostate, and colon cancer, and increases the apoptosis of cancer cells [13,14]. Recent studies also suggest that PuA could be used in the prevention and treatment of neurodegenerative diseases such as Alzheimer’s, Parkinson’s, and Huntington’s [15].

**Figure 1:**
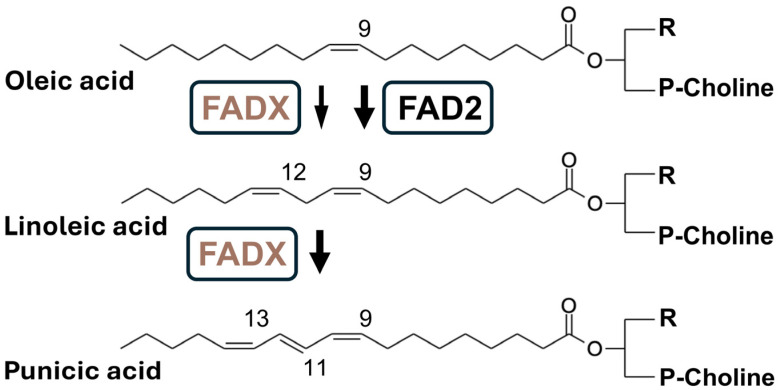
Biosynthesis of punicic acid (PuA). The oleic acid is converted into linoleic acid by the FAD2 desaturase. Linoleic acid is then transformed into PuA through the activity of fatty acid conjugase (FADX). This conversion occurs while the acyl moiety is esterified to the phosphatidylcholine molecule, creating conjugated double bonds at positions C11 and C13. PgFADX is a bifunctional enzyme, as it both catalyzes the production of PuA and converts oleic acid into linoleic acid, the precursor to PuA.

In pomegranate, the synthesis of PuA begins with the conversion of oleic acid (C18:1Delta9*cis*) into linoleic acid (C18:2Delta9*cis*,12*cis*) via the activity of FAD2 desaturase (Figure 1) [16,17]. PuA is synthesized from linoleic acid through the activity of the enzyme fatty acid conjugase (FADX), which is closely related in primary sequence to FAD2. This reaction occurs within the phosphatidylcholine molecule, leading to the formation of conjugated double bonds at positions C11 and C13. The *Punica granatum* FADX exhibits dual activity; it catalyzes the production of PuA and converts oleic acid into linoleic acid, the precursor of PuA. Finally, PuA is incorporated into triacylglycerol (TAG) lipid structures, where it is stored in the seed oil. Currently, the primary method for obtaining PuA is through the extraction of pomegranate seeds; however, this method has limitations due to the long growth cycle of the plants, climatic conditions, and their inherently low oil yield [18]. Despite the high concentration of PuA in pomegranate seed oil, its production is further challenged by significant variations in PuA content among different pomegranate cultivars [19]. These limitations underscore the need for alternative production methods, such as utilizing transgenic organisms, to ensure a stable and consistent supply of PuA.

The heterologous expression of the *PgFADX* in the *Saccharomyces cerevisiae* yeast has demonstrated that this yeast produces PuA in very limited amounts (0.8% of total fatty acids (TFA)) and only when grown in the presence of linoleic acid. Further efforts to enhance PuA production in recombinant *S. cerevisiae* cells (a deletion mutant in the transcription factor Snf2p, simultaneously expressing *PgFADX* with acyltransferases *PgPDAT* and *PgLPCAT*, together with the addition of 0.05% linoleic acid) led to a PuA accumulation reaching 3.37% of TFA [20].

Heterologous expression of the *PgFADX* in the *Arabidopsis thaliana* plant revealed that PgFADX inhibits the naturally occurring desaturase AtFAD2 [21]. An increased PuA accumulation was achieved in *A. thaliana* when *PgFADX* and *PgFAD2* were simultaneously expressed in seed lines with a significantly elevated level of linoleic acid. Recently, in a modified strain of *Brassica napus* with a higher oleic acid content, the co-expression of *PgFAD2* and *PgFADX* achieved 11.1% of PuA of TFA [22]. The PuA distribution in recombinant plants was high in phospholipids compared to pomegranate and low in TAG. These results suggest that lipid metabolism regulation, substrate availability, and intracellular channeling of PuA from phospholipids to TAG are essential processes for effective PuA accumulation in pomegranate seeds.

Initial attempts to produce PuA in the fission yeast *Schizosaccharomyces pombe* showed that, unlike the heterologous expression of *PgFAD2*, yeasts which express *PgFADX* exhibited slowed growth [23]. Recombinant *S. pombe* cells accumulated 38.7 mg/L of PuA, corresponding to 19.6% of PuA in TFA when *PgFADX* was expressed, and 34.3 mg/L of PuA, corresponding to 25.1% of PuA in TFA, in the case of the co-expression of *PgFADX* with *PgFAD2*. The dynamics of PuA accumulation in these heterologous strains were different and correlated with the growth defects of the yeast cultures tested. This difference is likely due to an inefficient deposition of PuA into TAG, leading to PuA accumulation in phospholipids, as well as in the free fatty acid fraction. Accumulation of PuA in membrane lipids and free fatty acids can lead to PuA lipotoxicity, potentially resulting from its interference with essential cellular processes [23].

In an effort to produce PuA by oleaginous organisms, recombinant strains of *Yarrowia lipolytica* were constructed [24]. The promoter optimization for *PgFADX* expression led to an improved PuA accumulation, from 0.9 to 1.8 mg/g of dry cell weight (DCW). The strain with the highest PuA production, expressing *PgFADX* under the control of a strong erythritol-inducible promoter, accumulated 36.6 mg/L of PuA. A recent study demonstrated that the yeast *Y. lipolytica* is a promising host for PuA production [25]. The resulting production strain with substantial multi-level genetic optimization, such as an increased linoleic acid content, multiple integrations of *PgFADX*, blocked beta-oxidation, and several pathway modifications for acyl-chain editing, produced 100.6 mg/L of PuA (4.77% of TFA) in shake flask conditions, and 3072.72 mg/L of PuA in a fermenter.

In this study, we constructed recombinant *R. toruloides* strains for PuA production without disrupting the synthesis of carotenoids, which can prevent lipid oxidation during the downstream processes of PuA isolation. The effects of three different promoters and two carbon sources were tested. In the best PuA-producing strains cultivated in media containing 6% glucose and 6% waste crude glycerol, PuA accumulation reached 105.77 mg/L (6.06 mg/g DCW, 1.32% of TFA) and 72.81 mg/L (3.56 mg/g DCW, 0.68% of TFA), respectively. The levels achieved were comparable to those recently reported for engineered *Y. lipolytica* strains in flask experiments, even without substantial metabolic pathway modifications. This suggests that recombinant *R. toruloides* is a more suitable oleaginous yeast for sustainable PuA production compared to *Y. lipolytica*. This study is the first, to our knowledge, to attempt the production of PuA-enriched single-cell oil using the recombinant red yeast *R. toruloides*.

## 2. Materials and Methods

### 2.1. Strains, Media and Cultivation

Strains used in this study are listed in Table 1. NEB 5-alpha *Escherichia coli* cells were used for plasmid construction. *Agrobacterium tumefaciens* EHA105 [26] was used for *A. tumefaciens*-mediated transformation (ATMT) of *Rhodotorula/Rhodosporidium toruloides*. *R. toruloides* IFO0880 (also known as APA2687 or NBRC0880) was a starting strain for all subsequent genetic modifications. *E. coli* and *A. tumefaciens* cells were cultivated at 37 °C and 30 °C in Luria Bertani (LB) medium (10 g/L tryptone, 5 g/L yeast extract, 10 g/L NaCl), respectively. To maintain the plasmids, the LB medium was supplemented with 50 mg/L kanamycin. *R. toruloides* was cultivated at 30 °C, 160 rpm in the YPD medium (20 g/L glucose, 20 g/L peptone, and 10 g/L yeast extract). Cell growth was estimated spectrophotometrically (Spectrophotometer, Biochrom Libra S2, Cambridge, UK) by measuring the OD at 600 nm. The yeast inoculum was prepared in 5 mL of the YPD medium in a microbial tube and an 18 h old inoculum was used for the inoculation of 50 mL production media MedA^+^ to OD of 0.5 in a 250 mL baffled Erlenmeyer flask. The production MedA^+^ growth medium was prepared by a modification of the MedA medium [27] to obtain a high C/N ratio leading to an increased accumulation of lipids in yeasts. The lipid production MedA^+^ medium consisted of 1.5 g/L yeast extract, 0.5 g/L NH_4_Cl, 7 g/L KH_2_PO_4_, 5 g/L Na_2_HPO_4_·12H_2_O, 0.1 g/L CaCl_2_, 1.5 g/L MgSO_4_·7H_2_O, 10 mg/L ZnSO_4_·7H_2_O, 0.6 mg/L FeCl_3_·6H_2_O, 0.07 mg/L MnSO_4_·H_2_O, and 0.04 mg/L CuSO_4_·5H_2_O, and the carbon source, at a concentration of 60 g/L, was either glucose (Slavus, Bratislava, Slovakia) or crude glycerol (Mikrochem, Pezinok, Slovakia). For the nitrate reductase promoter, NH_4_Cl was replaced by 0.8 g/L NaNO_3_ in a MedA^+^ medium. The preculture cells were cultivated in the 5 mL YPD inoculum and used to inoculate 50 mL of the production MedA^+^ medium containing NaNO_3_ instead of NH_4_Cl, as described above. Cells were cultivated at 30 °C and 160 rpm inside an orbital shaker (Innova 40, Hamburg, Germany) for 24 to 168 h depending on the experiment. DCW was measured gravimetrically. The residual glucose in cell-free supernatant was determined using the GlucCell glucose monitoring system (Chemglass Life Sciences, Vineland, NJ, USA), as previously reported [28].

### 2.2. Plasmid Construction, Transformation, and Verification

The *PgFADX* was codon-optimized (PQ281407) according to the codon preference of *R. toruloides* and synthetized by Generay Biotech (Shanghai, China). The terminator sequence of the plasma membrane H^+^-ATPase (T*_PMA1_*, PQ281408), the promoter sequences of glucose-6-phosphate isomerase (P*_PGI1_*, PQ281411), the nitrate reductase (P*_NAR1_*, PQ281410), and the plasma membrane H^+^-ATPase (P*_PMA1_*, PQ281409) were amplified from the genomic DNA of *R. toruloides* using the primer pairs listed in Table S1. The codon-optimized *PgFADX* sequence, and the promoter and the terminator sequences are listed in Table S2. To construct the plasmids pZPK-P*_PGI1_*-*PgFADX*-T*_PMA1_*-P*_GPD1_*-*HYG*-T*_NOS_*, pZPK-P*_NAR1_*-*PgFADX*-T*_PMA1_*-P*_GPD1_*-*HYG*-T*_NOS_*, and pZPK-P*_PMA1_*-*PgFADX*-T*_PMA1_*-P*_GPD1_*-*HYG*-T*_NOS_*, three fragments consisting of the corresponding promoter sequence, *PgFADX*, and T*_PMA1_* were assembled into the binary linearized vector pZPK-P*_GPD1_*-*HYG*-T*_NOS_* [29] using the Gibson assembly reaction (E5520S, New England Biolabs, Ipswich, MA, USA). The binary vector facilitates the random integration of genes of interest into the *R. toruloides* genome via the ATMT transformation protocol. The plasmids were amplified in *E. coli*, electroporated into *A. tumefaciens*, and then used to transform *R. toruloides* IFO0880 according to the ATMT, as described previously [30]. The transformants were resuspended in YPD and spread on YPD plates supplemented with 50 mg/L hygromycin B, 300 mg/L cefotaxime, and 300 mg/L carbenicillin to recover single clones. To verify the integration of the expected DNA fragment, *R. toruloides* colonies were subjected to colony-PCR with the primers listed in Table S1, according to the previously described method [29].

### 2.3. Lipid Extraction Procedure

Lipids were extracted using a method previously reported, with minor modifications [23]. Briefly, yeast cells (aliquots of OD 50—corresponding to approximately 10–13 mg of DCW) were collected by centrifugation, washed, and the cell pellets were frozen. The cells were suspended in 1 mL of a mixture of chloroform and methanol (2:1, *v*/*v*) containing the antioxidant butylated hydroxytoluene (BHT) at a final concentration of 0.01% and disrupted by FastPrep disintegrator (MP Biomedicals, Santa Ana, CA, USA) with glass beads (diameter 0.4 mm, 3 × 40 s at the highest speed, with 5 min cooling on ice between cycles) to obtain homogenates. For the thin layer chromatography (TLC), lipids were extracted from homogenates by chloroform/methanol/water (1:2:0.8, *v*/*v*), and subsequently, the proportion of the mixture was adjusted to 2:2:1.8 (*v*/*v*) at room temperature, according to the procedure of [31]. The organic phase containing the lipids was separated by centrifugation; the lipids were dried under a stream of N_2_, and the dry lipids were dissolved in a 100 μL mixture of chloroform and methanol (2:1, *v*/*v*) and BHT, prior to use.

### 2.4. Analytical Methods

For TLC analyses, an aliquot of lipid extract corresponding to 0.5 mg of DCW for neutral lipids and 3 mg of DCW for phospholipids was applied to Silica Gel 60 TLC plates (Merck, Darmstadt, Germany) using a Linomat 5 semiautomatic sample applicator (CAMAG Linomat 5, Muttenz, Switzerland). Neutral lipids were separated by a two-step TLC solvent system using a method described previously (first step: petroleum ether/diethyl ether/acetic acid, 70:30:2; second step: petroleum ether and diethyl ether, 49:1) [32]. Individual lipid spots were visualized by charring the plates, as previously reported [33]. Phospholipids were separated by the solvent system (chloroform/methanol/acetic acid/water, 75:45:3:1), as described previously [34]. Individual lipid spots were identified using lipid standards. The presence of PuA in individual lipids was determined by a densitometry scan at 276 nm (CAMAG TLC Scanner 3, Muttenz, Switzerland).

For fatty acid analysis, the total lipid homogenate, corresponding to approximately 10 mg DCW, was transmethylated with 5% Na-OCH_3_ in methanol. Fatty acid methyl esters (FAME) were then extracted using *n*-hexane, as described previously [17]. The analysis of FAME was performed by the injection of 1 μL aliquots into a gas chromatography (GC) apparatus (GC2010Plus, Shimadzu, Kyoto, Japan) equipped with a BPX70 capillary column (30 m × 0.25 mm × 0.25 µm, SGE Analytical Science, Melbourne, Australia), as described previously [23,35]. Individual FAMEs were identified by comparing them with authentic standards (C4−C24 FAME mixture, Supelco, Bellefonte, PA, USA). The quantification of individual fatty acids was conducted using tridecanoic acid methyl ester as an internal standard (Merck, Darmstadt, Germany). To determine the relative fatty acids content in TAG and phospholipids, the corresponding lipid spots were scraped off the TLC plate into glass tubes and transmethylated, as described above.

### 2.5. Fluorescence Microscopy

The *R. toruloides* wild type IFO0880 strain was cultured in a YPD medium or in a MedA^+^ medium containing 6% glucose or glycerol for 48 h and 72 h. The cell culture was diluted to OD = 2.0, harvested by centrifugation, washed once with a 50 mM Tris-HCl, pH 7.5, and suspended in 0.3 mL of 50 mM Tris-HCl, pH 7.5. LD540 (a stock solution of 50 mg/L in ethanol) was added to the final concentration of 1 mg/L, and the cells were incubated in the dark for 15 min at room temperature. A 3 µL drop of cell suspension was examined for the presence of the lipid droplets using a Leica DM5500 fluorescence microscope equipped with an HCX PL Fluotar 100× objective, and a Leica DFC340 FX digital camera (Leica Microsystems, Wetzlar, Germany). Signals were detected using the filter system Y3 for CY3 green. All images captured at each indicated time point used identical instrument settings and were processed using LAS 3.0 software (Leica Microsystems, Wetzlar, Germany).

### 2.6. Statistics and Reproducibility

All experiments were performed at least in duplicate. The data were expressed as means ± standard errors. All data analysis was performed by Excel 16.88 software (Microsoft, Redmond, WA, USA).

## 3. Results and Discussion

### 3.1. Promoter Selection, Strain Construction, and Screening of the Transformants Expressing PgFADX

**Figure 2:**
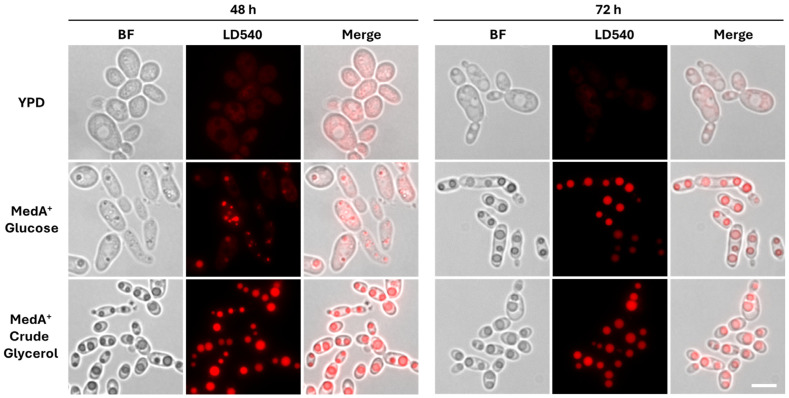
Fluorescence microscopy of *R. toruloides* wild type IFO0880 strain. Cells were grown in the rich medium (YPD) and in a lipid production medium (nitrogen-limited MedA^+^) supplemented with either 6% glucose or crude glycerol at 30 °C for indicated time. Cells were stained with LD540 to visualize the lipid droplets. Scale bar: 5 µm. Abbreviations: BF, bright field.

The strain IFO0880 natively accumulates lipids at high titers [8] and was, therefore, utilized for the engineering of PuA production. First, three plasmids expressing the *PgFADX* controlled by three different promoters on the pZPK-P*_GPD1_*-*HYG*-T*_NOS_* backbone were constructed. One of the promoters selected was a constitutive P*_PGI1_* promoter of the glucose 6-phosphate isomerase. This promoter was previously shown to be four times stronger than the promoter of the glyceraldehyde 3-phosphate dehydrogenase, P*_GPD1_*, when driving the expression of hygromycin resistance marker in cells supplemented with an increased concentration of hygromycin B [36]. In the effort to produce conjugated linolenic acid (CLNA) positional and geometric isomers in recombinant fission yeast, it was shown that a high accumulation of PuA and calendic acid influences cell growth [23,37]. Hence, the second promoter selected was an inducible promoter P*_NAR1_*, which drives nitrate reductase gene expression and is regulated by the nitrogen source [38]. The third promoter selected was P*_PMA1_*, which drives the plasma membrane proton-transporting ATPase [39] and is often used as a constitutive promoter in the model yeast *S. cerevisiae* [40]. The expression cassettes containing codon-optimized *PgFADX* under the control of the three different selected promoters were randomly integrated into the *R. toruloides* IFO0880 genome using the ATMT. After selection of the stable transformants, the presence of an insertion cassette in the genomic DNA was confirmed by PCR analysis. Nitrogen-limited MedA^+^ medium used to stimulate lipid formation in oleaginous yeast *Y. lipolytica* [41] was employed in this study. Lipid droplets were observed in living cells by staining with a lipid droplet-specific dye LD540 [42]. As shown in Figure 2, the *R. toruloides* IFO0880 cultured in a MedA^+^ medium showed enlarged lipid droplets compared to cells grown in a rich YPD medium. After 48 h of incubation, larger lipid droplets were observed in cells cultured in MedA^+^ medium with crude glycerol as the carbon source, although the lipid bodies were more abundant in cells grown in the glucose medium. At 72 h of incubation, the size of lipid droplets in cells grown in both media were comparable. Taken together, our results suggest that a MedA^+^ medium supplemented with glucose or crude glycerol could be a suitable medium for lipid production in *R. toruloides*.

**Figure 3:**
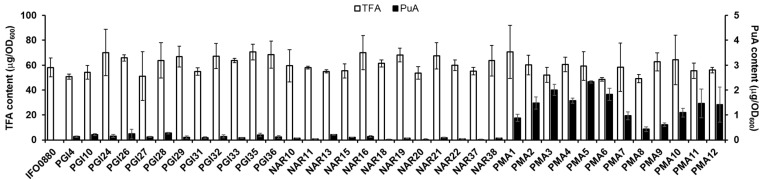
Screening of *R. toruloides* transformants for production of punicic acid (PuA). Cells were grown in the lipid production MedA^+^ medium containing 6% glucose at 30 °C for 72 h. Abbreviations: OD, optical density; TFA, total fatty acids.

Twelve randomly selected *PgFADX*-containing transformants for each promoter were screened for their ability to produce PuA. Transformants obtained by the ATMT usually have the expression cassette randomly integrated into the genome, which can influence cell growth and the expression of the desired gene from the cassette [43]. As shown in Figure 3, randomly picked *PgFADX*-containing transformants accumulated comparable levels of TFA per cell growth as wild type IFO0880. Successful production of PuA in all tested engineered recombinant strains was observed. Generally, a relatively higher content of PuA in TFA was observed for transformants expressing *PgFADX* under the control of the P*_PMA1_* promoter. The use of the P*_PMA1_* promoter enhanced the production of PuA and the best PuA-producing strain, PMA5, accumulated 8.6-fold and 11.1-fold more µg/OD of PuA compared to the best-producing PuA strains expressing the other two promoters, PGI28, and NAR13, respectively. This result confirms the importance of promoter choice for the production of PuA. It also validates the choice of multiple transformants as a suitable screening method of engineered strains when ATMT transformation is used to obtain transformants with random integration of the desired cassette.

### 3.2. Accumulation and Distribution of PuA in Production Media Containing Glucose

**Figure 4:**
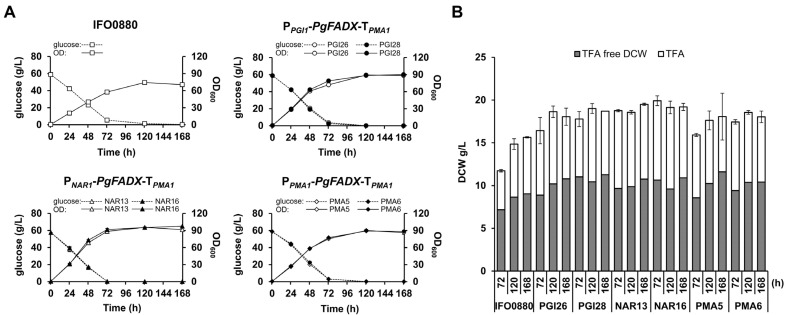
Flask characterization of IFO0880 (wild type strain), PGI26 and PGI28 (P*_PGI1_*-*PgFADX*-T*_PMA1_*), NAR13 and NAR16 (P*_NAR1_*-*PgFADX*-T*_PMA1_*), PMA5 and PMA6 (P*_PMA1_*-*PgFADX*-T*_PMA1_*) cultivated in MedA^+^ with 6% glucose as the carbon source. (**A**) Growth and glucose consumption, (**B**) dry cell weight (DCW) and total fatty acid (TFA) production. Gray bar area represents TFA-free biomass (g/L) and white area represents TFA (g/L).

It was previously shown that the production of CLNA isomers is a dynamic process [23,25,37]. Therefore, PuA-producing transformants were analyzed for the dynamics of PuA production in a time-dependent manner. Two transformants for each promoter were selected, namely PGI26 and PGI28, NAR13 and NAR16, and PMA5 and PMA6, which express *PgFADX* from P*_PGI_*, P*_NAR1_*, and P*_PMA1_* promoters, respectively. First, the growth, biomass yield, glucose consumption, and fatty acid production in the engineered strains were compared to the wild type strain IFO0880 (Figure 4 and Table 2). All the selected recombinant strains containing randomly integrated *PgFADX* expression cassettes grew comparably well, with a slight increase in OD and DCW compared to the wild type strain. Most of the glucose was consumed after 72 h of cultivation. All strains accumulated, on average, 40–50% of TFA in biomass. The fatty acid profile of the wild type strain remained stable during the cultivation periods of 72 h, 120 h, and 168 h (Table S3). A substantial difference was observed in the ratio of monounsaturated to polyunsaturated fatty acid (MUFA/PUFA) for the PuA-producing recombinant strains. The expression of *PgFADX* resulted in an increased relative content of oleic acid (C18:1) and decreased levels of linoleic acid (C18:2) and α-linolenic acid (C18:3). These observed changes correlated with the elevation of PuA levels in the recombinant cells. This result suggests that the PgFADX might compete for the C18:1 substrate with the activity of the endogenous FAD2 in the engineered strains, similar to what was observed in recombinant *A. thaliana* plants [21].

The highest relative content of PuA in TFA was 1.3% in the PMA6 strain containing the P*_PMA1_* promoter, which was much higher than in the strains with *PgFADX* expressed from the P*_PGI_* and P*_NAR1_* promoters (Table 2). When productivity is taken into consideration, the best PuA-producing strain PMA6 reached 105.8 mg/L PuA and 6.1 mg/g DCW at 30 °C after 72 h (Table 2). Similar results were obtained with prolonged cultivation times of 120 h and 168 h, suggesting that the PuA level is stable over time in *R. toruloides* cells. The expression under P*_PGI_* and P*_NAR1_* promoters showed a 10-fold and 9-fold lower production of PuA, respectively, compared to the P*_PMA1_* promoter.

It is worth emphasizing that the PuA titer was 5.4-fold higher than the 19.6 mg/L titer obtained in recombinant *S. pombe* over-expressing *PgFADX* from the strong inducible *NMT1* promoter [23], and 2.9-fold higher than the 36.6 mg/L titer in the recombinant obese *Y. lipolytica* strain expressing *PgFADX* from the hybrid inducible *pEYK1 4AB-coreTEF* promoter [24]. It is proposed that the main limitation in PuA accumulation is the inefficient flux of PuA from phospholipids to TAG in recombinant yeasts. This hypothesis was proven in the recombinant model yeast *S. cerevisiae* [20] and more recently in the recombinant oleaginous yeast *Y. lipolytica*, which can accumulate increased levels of PuA with extensive genetic optimization, including enhanced C18:2 supply, expression of multiple copies of *PgFADX*, interruption of *β*-oxidation, and over-expression of genes involved in acyl-editing and glycerol-3-phosphate synthesis pathways [25].

To sum up, our results demonstrated that the recombinant oleaginous red yeast *R. toruloides*, expressing *PgFADX* from a P*_PMA1_* promoter, is capable of producing a PuA titer comparable to the 100.56 mg/L recently reported for the engineered oleaginous yeast *Y. lipolytica* in shake flask fermentation. The relative level of PuA in engineered *R. toruloides* is not high; therefore, it is expected that further optimization of the metabolic pathways, including but not limited to the carbon and C18:2 supply, enhanced PuA synthesis, and PuA channeling to TAG lipid structures could lead to a significant increase in PuA titer.

CLNA isomers are preferentially synthesized through biotransformation of linoleic acid esterified to phosphatidylcholine in native producers, and then, very efficiently channeled from membrane phospholipids to lipid storage depots, lipid droplets, in the form of TAG [21,44]. However, the precise mechanism of CLNA isomer channeling is still not well understood and is currently under investigation. Previous studies have demonstrated a significant difference in the relative content of PuA in TAG and phosphatidylcholine lipid structures between pomegranate seeds, which naturally produce PuA, and the seeds of transgenic plants [21]. Therefore, the relative content of PuA in TAG and phospholipids was examined.

**Figure 5:**
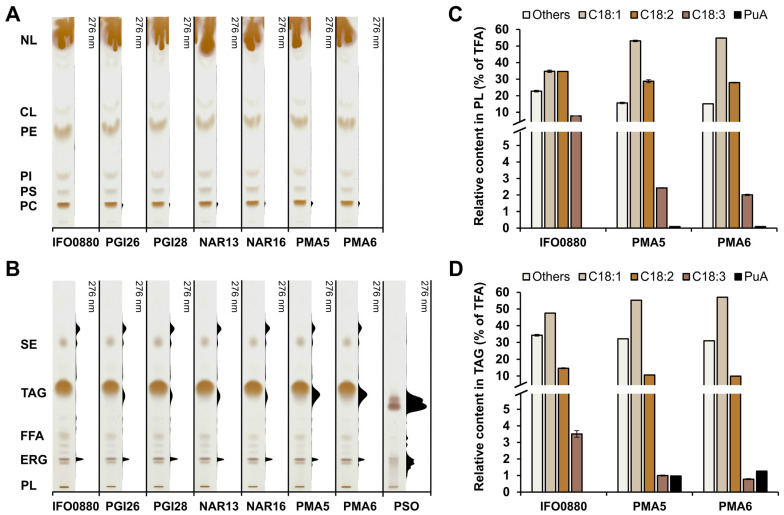
Representative TLC chromatography scans of (**A**) polar lipids (phospholipids) and (**B**) neutral lipids (NL) extracted from *R. toruloides* wild type IFO0880 strain and strains expressing *P. granatum* fatty acid conjugase (*PgFADX*) grown for 168 h in MedA^+^ supplemented with 6% glucose. A total of 30 µg of pomegranate seed oil (PSO) was used as a control. Relative fatty acid content is represented in (**C**) phospholipids and (**D**) triacylglycerols. Abbreviations: CL, cardiolipin; ERG, ergosterol; FFA, free fatty acids; PC, phosphatidylcholine; PE, phosphatidylethanolamine; PI, phosphatidylinositol; PL, phospholipids: PS, phosphatidylserine; SE, steryl esters; TAG, triacylglycerols; TFA, total fatty acids.

In order to characterize the distribution of PuA in individual lipid classes, the presence of conjugated double bonds in the PuA structure, which allows its detection under UV light, was utilized [45]. First, total lipid extracts of *R. toruloides* were separated on TLC plates and the phospholipids were analyzed. With this approach, PuA was mainly detected in the phosphatidylcholine (Figure 5A), which is reported to be the primary place for CLNA isomer synthesis. Second, the total lipid extracts were loaded on a TLC plate and the plate was developed in conditions favoring the separation of neutral lipids. The majority of PuA was detected in TAG (Figure 5B). The presence of PuA in steryl esters could not be verified due to possible interference from the signal of some sterol molecules containing conjugated double bonds. Furthermore, the PuA content in the free fatty acids fraction was negligible. This is in contrast with the presence of PuA or calendic acid in lipid extracts from recombinant yeast strains accumulating substantial amounts of these fatty acids in free form [18,24,37].

It is worth noting that, although the relative amount of PuA in engineered *R. toruloides* strains is not high, the UV signal indicates that PuA is predominantly distributed in TAG lipid structures (Figure 5A,B). This result suggests that, in engineered *R. toruloides* strains, the PuA is efficiently channeled from the site of synthesis (phosphatidylcholine) to the TAG lipid structures. However, this might be due to the very high ratio of TAG to phospholipids in engineered strains under tested conditions. To further analyze the distribution of PuA in lipid fractions in the two best PuA-producing strains, PMA5 and PMA6, the relative fatty acid content in phospholipids and TAG was determined (Figure 5C,D). In both strains, the relative content of C18:1 increased in the lipid fractions examined compared to the wild type strain, while the relative content of C18:3 showed an almost 4-fold decrease. The PuA content in both strains was <0.1% and approximately 1% in the phospholipids and TAG fractions, respectively. The high enrichment of PuA in the TAG lipid structures and, at the same time, the high titers of PuA with a minimal requirement for genome modification suggests that the red yeast *R. toruloides* is a more suitable oleaginous yeast for PuA production than the yeast *Y. lipolytica*.

### 3.3. Accumulation and Distribution of PuA in Production Media Containing Crude Glycerol

In our initial experiments, the accumulation of enlarged lipid droplets was observed in the *R. toruloides* wild type IFO0880 strain, grown in a MedA^+^ medium supplemented with 6% crude glycerol (Figure 2). The response of promoters may vary depending on a carbon source impacting the final titer of the desired product [46,47] or the PuA production efficiency. To determine the effect of the carbon source, crude glycerol was evaluated with all three promoters.

**Figure 6:**
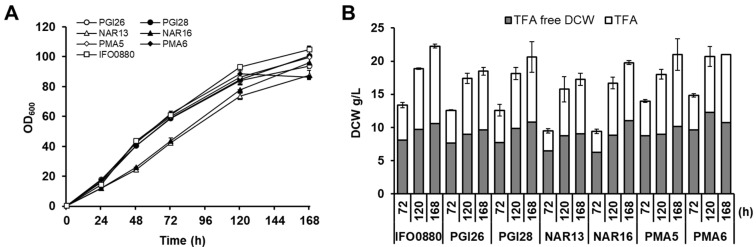
Flask characterization of IFO0880 (wild type strain), PGI26 and PGI28 (P*_PGI1_*-*PgFADX*-T*_PMA1_*), NAR13 and NAR16 (P*_NAR1_*-*PgFADX*-T*_PMA1_*), PMA5 and PMA6 (P*_PMA1_*-*PgFADX*-T*_PMA1_*) cultivated in MedA^+^ with 6% crude glycerol as the carbon source. (**A**) Growth, (**B**) biomass—dry cell weight (DCW) and total fatty acid (TFA) production. Gray area represents TFA-free biomass (g/L) and white area represents TFA (g/L).

The recombinant strains producing the highest PuA titer for all three promoters were examined for growth, TFA accumulation, PuA content, and its distribution on crude glycerol. The procedure was similar to that used with the MedA^+^ medium supplemented with glucose. As shown on Figure 6A, the growth of PuA-producing *R. toruloides* strains was comparable to the growth of the wild type IFO0880. Compared to the glucose-containing medium (Figure 4), the growth in crude glycerol was slightly slower, but after 120 h, the cells reached a similar OD. The biomass yield and lipid accumulation increased with cultivation time (Figure 6B) and strains accumulated, on average, approximately 40–50% of TFA in biomass at the 120 h and 168 h time points (Table 3). This result confirms that crude glycerol is a suitable low-cost carbon source for microbial lipid production using *R. toruloides*. Similar to the glucose medium, the relative content of fatty acids in the wild type strain remained stable during the cultivation periods of 72 h, 120 h, and 168 h (Table S4). However, the relative content of PUFA decreased in the MedA^+^ supplemented with glycerol compared to the glucose medium.

In general, the amount of PuA increased with the cultivation time (Table 3). In comparison to the glucose medium, the PuA titer significantly increased in strains with *PgFADX* expressed from the P*_NAR1_* promoter when cultivated on a low-cost crude glycerol medium. The highest relative level of PuA was 0.9% of TFA at 72 h in the PMA5 strain containing the P*_PMA1_* promoter, which was again much higher than in the strains with *PgFADX* expressed from the P*_PGI_* and P*_NAR1_* promoters. When productivity is taken into consideration, the best PuA-producing strain, PMA5, achieved a total PuA titer of 72.8 mg/L and 3.6 mg/g DCW at 30 °C after 168 h in shake flask conditions. It should be noted that most of the strains did not stop growing in the crude glycerol-containing medium, which could impact the results, including the PuA titer, when compared to the glucose-containing medium. Nevertheless, the level of PuA obtained in the PMA5 strain is approximately two-fold higher than what has been reported for the engineered *S. pombe* [23], and *Y. lipolytica* [24] strains. However, it is about one-third lower than the titers reported in a recent study on recombinant *Y. lipolytica*, which achieved higher titers through significant optimization of the metabolic pathways grown with glucose as a carbon source in shake flask conditions [25].

**Figure 7:**
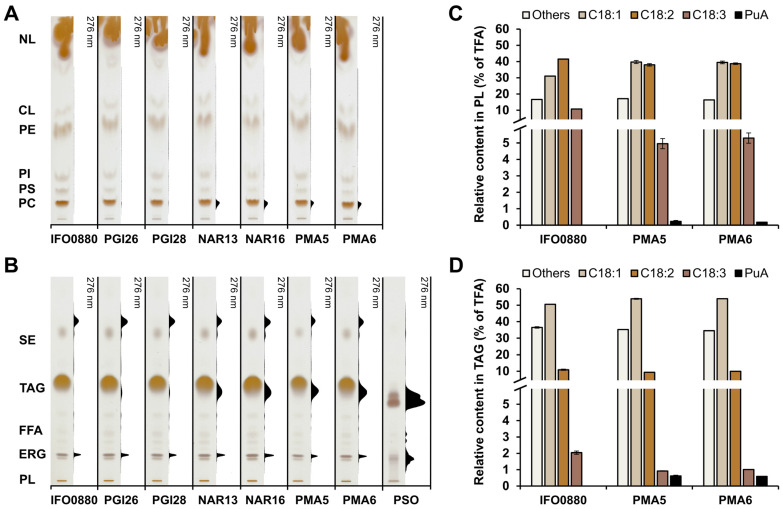
Typical TLC chromatography scans of (**A**) polar lipids (phospholipids) and (**B**) neutral lipids (NL) extracted from *R. toruloides* wild type IFO0880 strain and strains expressing *P. granatum* fatty acid conjugase (PgFADX) grown for 168 h in MedA^+^ supplemented with 6% crude glycerol. A total of 30 ug of pomegranate seed oil (PSO) was used. Relative fatty acid content in (**C**) phospholipids and (**D**) triacylglycerols. Abbreviations: CL, cardiolipin; ERG, ergosterol; FFA, free fatty acids; PC, phosphatidylcholine; PE, phosphatidylethanolamine; PI, phosphatidylinositol; PL, phospholipids: PS, phosphatidylserine; SE, steryl esters; TAG, triacylglycerols; TFA, total fatty acids.

To examine the distribution of PuA in individual lipid classes, the total lipids were separated on TLC plates and the detection of PuA under UV light was used, as described above. Similarly, as with the glucose-containing medium, the signal for PuA was mainly detected in phosphatidylcholine (Figure 7A) and TAG lipid structures (Figure 7B). The signal for PuA in the free fatty acid fraction was negligible. Next, the relative contents of PuA in phospholipid and TAG fractions in the two best PuA-producing strains, PMA5 and PMA6, were analyzed (Figure 7C,D). The PuA content in both strains was approximately 0.2% and 0.6% in the phospholipids and TAG fractions, respectively. The enrichment of PuA in the single-cell oil, which is comprised of TAG lipid structures, was not as high as in the glucose medium. However, since the TAG are the predominant lipid molecules in *R. toruloides*, grown under nitrogen-limited conditions, with glycerol as the carbon source, the majority of PuA is stored in TAG lipid structures. Our results confirmed that the medium with a low-cost carbon source is suitable for the high production of PuA by the recombinant red yeast *R. toruloides*.

The price of media components, mainly carbon and nitrogen sources, is a critical factor in any biotechnological process [48,49]. *R. toruloides* exhibits greater versatility than *Y. lipolytica* in assimilating low-cost carbon substrates, including crude glycerol, lignocellulosic hydrolysates, and molasses [2]. This versatility significantly enhances its potential use in various biotechnological applications. Moreover, *R. toruloides* can accumulate more lipids from crude glycerol than from pure glycerol, without being negatively affected by the impurities present in crude glycerol [50]. Converting a low-cost carbon source into a value-added product, such as PuA, could significantly reduce the upstream expenses, thereby making the overall production process more economically viable. An additional advantage of *R. toruloides* is its natural production of carotenoids, which act as antioxidants and are widely used in food, pharmaceuticals, and cosmetics [3,4]. It has been demonstrated that carotenoids can effectively inhibit lipid peroxidation [51]. Therefore, carotenoids, which co-purify with lipids during the lipid extraction from engineered *R. toruloides* cells under our experimental conditions, could prevent PuA oxidation of PuA-enriched single-cell oil as the antioxidative capacity of carotenoids has been demonstrated for long-chain PUFA-rich oil [52].

Another notable difference between the two oleaginous yeasts is that *R. toruloides* naturally synthesizes C18:3, unlike *Y. lipolytica*. It is well documented that the level of unsaturation in PUFA correlates with increased membrane fluidity, elasticity, and flexibility [53]. Therefore, the presence of unusual PUFAs, such as PuA, in membrane lipids may influence cellular membrane properties, including fluidity, integrity, permeability, and sensitivity to oxidative stress. This could trigger a cellular response that enhances the channeling of PuA into TAG lipid structures. It has been shown that flax long-chain acyl-CoA synthetase (LACS8A) and diacylglycerol acyltransferase (DGAT2-3), which are involved in TAG accumulation, exhibit greater specific enzymatic activity towards C18:3 and C18:3-CoA, respectively, leading to enrichment of TAG structures with C18:3 [54]. Therefore, it can be hypothesized that homologous enzymes from *R. toruloides* may have an enhanced substrate preference for PuA and PuA-CoA compared to analogous enzymes from *Y. lipolytica*, which naturally does not synthetize C18:3. Considering these factors, along with the high accumulation of PuA in engineered *R. toruloides* cells, it is evident that *R. toruloides* may have greater biotechnological potential for PuA production than *Y. lipolytica*.

## 4. Conclusions

For the first time, to our knowledge, the low-cost substrate, crude glycerol, was used for the production of a single-cell oil enriched in PuA using the recombinant red yeast *R. toruloides*. The use of a promoter driving the expression of plasma membrane ATPase to express *PgFADX* improved the overall productivity of the recombinant strain. The high PuA titer and its enrichment in TAG lipids, together with the minimal genome modification, suggests that the red yeast *R. toruloides* is a more suitable host for PuA production compared to *Y. lipolytica*. In addition, the red yeast naturally synthesizes carotenoids, which possess nutritional value, and can prevent the oxidation of CLNA and its positional and geometric isomers during the downstream processing of lipids. With the further optimization of the metabolic pathways, and growth medium formulation, further improvements of red yeast *R. toruloides* for sustainable PuA production are expected.

## Figures and Tables

**Table 1:** Strains used in this study.

Strain	Characteristics	Source
***Escherichia coli***		
NEB 5-alpha	*fhuA2*Δ*(argF-lacZ)U169 phoA glnV44 Φ80*Δ*(lacZ)M15 gyrA96 recA1 relA1 endA1 thi-1 hsdR17*	NEB #C2987
***Agrobacterium tumefaciens***		
EHA105	derivative of A281 (A136/pTiBo542)	Skerker J.M., UC Berkeley
EHA105-PGI1-PgFADX	EHA105/pZPK-P*_PGI1_*-*PgFADX*-T*_PMA1_*-P*_GPD1_*-*HYG*-T*_NOS_*	This study
EHA105-NAR1-PgFADX	EHA105/pZPK-P*_NAR1_*-*PgFADX*-T*_PMA1_*-P*_GPD1_*-*HYG*-T*_NOS_*	This study
EHA105-PMA1-PgFADX	EHA105/pZPK-P*_PMA1_*-*PgFADX*-T*_PMA1_*-P*_GPD1_*-*HYG*-T*_NOS_*	This study
***Rhodotorula toruloides***		
IFO0880	MAT A2	Skerker J.M., UC Berkeley
PGI4–PGI36	IFO0880/P*_PGI1_*-*PgFADX*-T*_PMA1_*-P*_GPD1_*-*HYG*-T*_NOS_* cassette	This study
NAR10–NAR38	IFO0880/P*_NAR1_*-*PgFADX*-T*_PMA1_*-P*_GPD1_*-*HYG*-T*_NOS_* cassette	This study
PMA1–PMA12	IFO0880/P*_PMA1_*-*PgFADX*-T*_PMA1_*-P*_GPD1_*-*HYG*-T*_NOS_* cassette	This study

**Table 2:** Fatty acid accumulation in strains cultivated in MedA^+^ medium containing 6% glucose.

Strain	Promoter for *PgFADX*	Time (h)	TFA (g/L)	TFA/DCW (%)	PuA (% of TFA)	PuA (mg/g DCW)	PuA (mg/L)
		72	4.55 ± 0.17	38.81 ± 0.97	-	-	-
**IFO0880**		120	6.20 ± 0.02	41.80 ± 1.86	-	-	-
		168	6.61 ± 0.38	42.22 ± 2.20	-	-	-
		72	7.54 ± 0.08	46.09 ± 3.85	0.13 ± 0.00	0.59 ± 0.03	9.74 ± 0.49
**PGI26**	P*_PGI1_*	120	8.44 ± 0.62	45.23 ± 1.67	0.12 ± 0.00	0.55 ± 0.02	10.35 ± 0.71
		168	7.26 ± 1.36	40.46 ± 9.80	0.12 ± 0.00	0.49 ± 0.12	8.70 ± 1.74
		72	6.76 ± 0.67	37.97 ± 1.89	0.10 ± 0.00	0.37 ± 0.00	6.65 ± 0.40
**PGI28**	P*_PGI1_*	120	8.57 ± 0.71	45.05 ± 2.36	0.10 ± 0.00	0.43 ± 0.02	8.21 ± 0.66
		168	7.43 ± 1.02	39.71 ± 5.44	0.09 ± 0.00	0.37 ± 0.05	6.90 ± 0.90
		72	9.10 ± 0.55	48.50 ± 2.58	0.13 ± 0.00	0.62 ± 0.01	11.62 ± 0.09
**NAR13**	P*_NAR1_*	120	8.69 ± 0.27	46.81 ± 1.95	0.13 ± 0.01	0.61 ± 0.03	11.42 ± 0.61
		168	8.75 ± 0.55	44.84 ± 2.54	0.13 ± 0.00	0.59 ± 0.05	11.42 ± 1.03
		72	9.27 ± 0.22	46.53 ± 0.24	0.07 ± 0.00	0.33 ± 0.00	6.59 ± 0.09
**NAR16**	P*_NAR1_*	120	9.53 ± 0.10	49.86 ± 2.45	0.07 ± 0.00	0.36 ± 0.03	6.97 ± 0.29
		168	8.29 ± 0.65	43.16 ± 2.44	0.07 ± 0.00	0.31 ± 0.03	6.04 ± 0.61
		72	7.35 ± 0.51	46.16 ± 3.71	0.97 ± 0.11	4.45 ± 0.13	70.91 ± 2.86
**PMA5**	P*_PMA1_*	120	7.39 ± 0.53	42.09 ± 5.66	0.98 ± 0.01	4.14 ± 0.62	72.70 ± 6.34
		168	6.46 ± 2.10	35.26 ± 6.29	0.96 ± 0.01	3.37 ± 0.58	61.63 ± 19.63
		72	8.02 ± 0.58	45.98 ± 2.65	1.32 ± 0.06	6.06 ± 0.07	105.77 ± 2.81
**PMA6**	P*_PMA1_*	120	8.17 ± 0.17	44.05 ± 1.41	1.27 ± 0.03	5.60 ± 0.30	103.90 ± 4.48
		168	7.63 ± 0.74	42.39 ± 5.67	1.25 ± 0.02	5.28 ± 0.80	95.00 ± 10.78

Abbreviations: DCW, dry cell weight; PuA, punicic acid; TFA, total fatty acids.

**Table 3:** Fatty acid accumulation in strains cultivated in MedA^+^ medium containing 6% crude glycerol.

Strain	Promoter for *PgFADX*	Time (h)	TFA (g/L)	TFA/DCW (%)	PuA (% of TFA)	PuA (mg/g DCW)	PuA (mg/L)
		72	5.26 ± 0.15	39.40 ± 0.12	-	-	-
**IFO0880**		120	9.19 ± 0.21	48.73 ± 0.79	-	-	-
		168	11.71 ± 1.12	52.57 ± 4.33	-	-	-
		72	4.92 ± 0.08	39.11 ± 0.35	0.04 ± 0.00	0.17 ± 0.00	2.10 ± 0.01
**PGI26**	P*_PGI1_*	120	8.43 ± 0.97	46.38 ± 0.50	0.03 ± 0.00	0.15 ± 0.00	2.76 ± 0.29
		168	8.84 ± 1.32	49.67 ± 2.83	0.03 ± 0.00	0.16 ± 0.01	2.93 ± 0.38
		72	4.85 ± 0.35	38.53 ± 0.06	0.04 ± 0.01	0.14 ± 0.01	1.73 ± 0.01
**PGI28**	P*_PGI1_*	120	8.31 ± 0.74	43.18 ± 1.89	0.03 ± 0.01	0.11 ± 0.01	2.14 ± 0.09
		168	9.82 ± 2.71	49.70 ± 4.23	0.03 ± 0.01	0.12 ± 0.01	2.44 ± 0.57
		72	3.00 ± 0.03	31.65 ± 1.50	0.14 ± 0.01	0.43 ± 0.02	4.05 ± 0.03
**NAR13**	P*_NAR1_*	120	6.98 ± 1.12	41.43 ± 2.18	0.14 ± 0.01	0.58 ± 0.08	9.70 ± 0.67
		168	8.20 ± 1.99	50.20 ± 4.98	0.26 ± 0.04	1.26 ± 0.03	20.44 ± 2.48
		72	3.16 ± 0.03	33.71 ± 0.90	0.09 ± 0.01	0.29 ± 0.04	2.68 ± 0.25
**NAR16**	P*_NAR1_*	120	7.88 ± 1.68	42.08 ± 0.67	0.13 ± 0.01	0.53 ± 0.01	9.91 ± 1.87
		168	8.79 ± 1.60	48.90 ± 1.10	0.29 ± 0.02	1.40 ± 0.07	24.98 ± 2.69
		72	5.22 ± 0.25	37.33 ± 1.14	0.89 ± 0.08	3.33 ± 0.21	46.54 ± 2.13
**PMA5**	P*_PMA1_*	120	9.06 ± 0.97	48.95 ± 1.56	0.69 ± 0.06	3.35 ± 0.19	61.74 ± 1.18
		168	10.86 ± 1.96	52.83 ± 1.64	0.68 ± 0.06	3.56 ± 0.19	72.81 ± 7.12
		72	5.17 ± 0.11	34.88 ± 0.12	0.75 ± 0.00	2.63 ± 0.01	38.94 ± 0.86
**PMA6**	P*_PMA1_*	120	8.46 ± 0.13	41.64 ± 2.68	0.60 ± 0.00	2.48 ± 0.16	50.43 ± 0.75
		168	10.27 ± 0.89	47.96 ± 2.99	0.61 ± 0.01	2.92 ± 0.10	62.44 ± 3.56

Abbreviations: DCW, dry cell weight; PuA, punicic acid; TFA, total fatty acids.

## Data Availability

The original contributions presented in the study are included in the article/Supplementary Materials, further inquiries can be directed to the corresponding author.

## Supplementary Materials

The following supporting information can be downloaded at: https://www.mdpi.com/article/10.3390/jof10090649/s1, Table S1: Primers used in this study; Table S2: Promoter, codon-optimized *PgFADX*, and terminator sequences; Table S3: Fatty acid composition of wild type strain IFO0880 and *PgFADX*-expressing strains cultivated in MedA+ medium containing 6% glucose for 72 h, 120 h, and 168 h; Table S4: Fatty acid composition of wild type strain IFO0880 and *PgFADX*-expressing strains cultivated in MedA+ medium containing 6% crude glycerol for 72 h, 120 h, and 168 h.
