# Supplementary material for: The Plasma Membrane H+-ATPase Promoter Driving the Expression of FADX Enables Highly Efficient Production of Punicic Acid in Rhodotorula toruloides Cultivated on Glucose and Crude Glycerol
Source: J Fungi (Basel). 2024 Sep 13;10(9):649. doi: 10.3390/jof10090649 (PMC11433134; doi:10.3390/jof10090649)
Supplement: Supplementary file 1 [file jof-10-00649-s001.zip › jof-3186067-supplementary.pdf]

Supplementary Material

The plasma membrane H^+-ATPase promoter driving the expression of *FADX* enables highly efficient production of punicic acid in *Rhodotorula toruloides* cultivated on glucose and crude glycerol.

Table S1. Primers used in this study.

Primer	Sequence (5′-3′)	Description
pZPK-ERI-pPGI-F2	gaattaacgccgaattgaattcGCCGTCTGCCACTTTATCGTC	Amplification of P_{PGI1} promoter
pPGI-PgFADX-Rt-R2	gtgccgtccgcgcccatGGTTCGTAGCGTGGTGAGTGAGAA	
pZPK-ERI-pNar1-4-F	gaattaacgccgaattgaattcCAACGTCGGCCCGCCTT	Amplification of P_{NAR1} promoter
pNar1-PgFADX-Rt-R	gtgccgtccgcgcccatGTTCGTGGGTCGTTCTTCTGGG	
pZPK_ERI_pPMA1_F	gaattaacgccgaattgaattcCGATACGAGCAAAGAAGCGGC	Amplification of P_{PMA1} promoter
pPMA1_PgFADX_Rt_R	gtgccgtccgcgcccatGGCCGAGTGTTGCGCAGAG	
PgFADX_Rt_F	ATGGGCGCGGACGGC	Verification of *PgFADX* integration
Rt_tPMA1_R1	ATATGCGAAGGCGAGAGCGAG	

Table S2. Promoter, codon optimized *PgFADX*, and terminator sequences.

Name (NCBI accession number)	Sequence (5′-3′)
$\mathbf{P}_{\mathit{PGI1}}$ (PQ281411)	gccgtctgccactttatcgtcgcgggctgctcgacggccttgtcgcaggacaacgaggcgggtcagaaggtgttgattgggttcgtgtgtgtgtttatcgcgttctttgc<br>ggcgacttggtgaggcctgttctttccgcctttcgcgagaacagcagactgacgacttcgcttcaccaggggtccaatcgcatgggtcgtgtacgctaccactcctcgc<br>attctccgcgttgagctgagctgattcgggcttgctcgcagcacgagcgaaatctacgcgacggcgactcgggcgaaacagatgtcgatgtctgtcgcttccaactg<br>gctcttcagtgcgtcgtctctccctccattcctccctccacgcttcacctcagctgaacgaaccgctggacgcgcgcagatttcggtatcggttacgcgactccttgttcg<br>tctcctccccttttccgctcttcaggccgagagctgaccagccttccatgcgcgcagatctcgtcaatactggcaaaggaaacgcgggtcttcagggcaaggtgttcttc<br>atcgtgcgtcccgacccgttcttgctccctctttcgctcatgcggagggatgagtttgctgacgaggtatatgaatcgcgcagtggggcggtgtatcgtgcatcgcgatt<br>ctgtttgtgtacttgtgcgtcactcccttccccgccgcgctgcgacaaagcggcaggctgacgaccgacttgtacagctgtattcccgagaccaagggtctctcgctcg<br>aacaaagtccgccgcccattctgcctcgcacgcgttgtcgaatgaagtcactgacgcttgcgctaccgcagtcgacatcttataccgcctaaccggcaacgcgatcc<br>gggcgaccaagatccgccgcgaactgtgcgttccgctgtccctcctgctctccgcccatgtactgatgtcgtttctcgcctgcgcgcagcctcgagcgcgacatccaa<br>gacgaggacaccgccgcttacttctacgccttcaaggagcaacacctcggctcctcccttcacgagctcgagcagcaccaaggctcttcctccgacccgcagcaac<br>cgcagcatcaccaagtccagctgcagcaggacgagctcgcaccggtcgagagtccagcggtcaaaatgcgggacttgaacgaggcgtgaaggaccgcggacg<br>ggttgtacgacttcgcaggcgcatagactaacggggacggcttagacgcacactagacatagatctctcctctcgcatagacggctcgctgcccttgttgtagctcgta<br>cggttgctctcgtcggaattcgaggatgttgccctcttgacctcgcctgatcccgttcggtccagcgttggcgtcaacaagaggatatactggaccgctaggaattgca<br>agcaagcccatcggagcgacttgctgaatgacgccctcatcgggcccactcgctcgtcgctttcgctctcttctcactcaccacgctacgaacc
$\mathbf{P}_{\mathit{NAR1}}$ (PQ281410)	caacgtcggcccgccttgtcccaggtcgccgactgtcctatcgcaacactgcaactgtagcgctgttcgaaccggagaccttgcggcgcgatccgagggcaaagtc<br>gtgcatttgcgaaacggtatccgctcgaagggctcacgcgctcacgatagacctcgaccggcctcggacttggcgccagggtcgcttccgatgacggacagcaact<br>ctggctctgggcgtcgtcgctgctgcgatccactttgcgggtccccttatcgcgactgctggatccgagattggcggtatctttcgctcgctggtcttgttggacagctg<br>gacagcctctgcagcgtcgaagcgacgtcgataaactccagcgacacgctcacaatcccagaagaacgacccacgaac
$\mathbf{P}_{\mathit{PMA1}}$ (PQ281409)	cgatacgagcaaagaagcggctcgagagcgagacgggatcccctgctgtcaaattccgatccgaaccaccgacaagccgacgaaagacggccggccggcgga<br>cggcacacgagcgggaagggcgcaaagaactcagcgcgggtccgcctcgcgcttctgctgtttttggggagtaagttagtgagtggtcgggatgatcgcttcgagg<br>aaaagatagcgagtctagtctaggtcgaggagcaggagcaggagcagagcagggagaggaggcgggacgaggagcgcgagtctcctctctggtcgttccggcc<br>ggatccgcggagtatgatgctattgctgagttagccagcccgagccaagggaggctgtgagtgcacgagaagagcgcaaccaacggagcacagtgagcgcttgc<br>ttgagcgaaaaggacgagaagagcgagaggggtttcgctcgcgccgtctgttgccccgggatccgcgttatctatcgcagcagtgggaagcgagaggagagtgc<br>actctcgagcaggagtgagagagtggcgcagctgagagagagagtgcgagggagagtgaactgtgcccgagtccctcgcgccgaagtgctgagtccgaggagt<br>ggctccgtcatggcgaggtgagagagctggggccagcgtacagtagtgtgcgaaactgcgaagtgagggcggggtgcgaaaatgtgcgagggagagagtccgt<br>ccgccgagcgcgggtgcgctgggggtcgaagaggaggtcggcgtcggcgcgagctgagcgagacgggtgctgctctttctctctcttgctcttgttcttgactcc<br>agaagagcgtctcccactctttctgaaccccgtacgaaagagcaaggcactcgagcccaacttcggcctcgcccgcatccagccagccagcccgcctcgctctct<br>ctctttccctctttctcttcttcttcttcacccgacaactctgcgcaacactcggcc

***PgFADX* (codon optimized)** (PQ281407)	ATGGGCGCGGACGGCACCATGTCGCCGGTCCTCACGAAGCGCCGCCCGGACCAGGAGATCAACAAGCTCGACATCAAGCCGAACCACGAGGTCGACATCGCCCGCCGCGCCCCCACTCCAAGCCCCCGTTCACGCTCTCGGACCTCCGCTCGGCCATCCCGCCCCACTGCTTCCACCGCTCGCTCCTCATGTCGTCGTCGTACCTCATCCGCGACTTCGCCCTCGCCTTCCTCTTCTACCACTCGGCGGTCACGTACATCCCCCTCCTCCCGAAGCCGCTCGCCTGCATGGCGTGGCCGGTCTACTGGTTCCTCCAGGGCTCGAACATGCTCGGCATCTGGGTCATCGCGCACGAGTGCGGCCACCAGGCGTTCTCGAACTACGGCTGGGTCAACGACGCGGTCGGCTTCTTCCTCCACACGTCGCTCCTCGTCCCCTACTTCCCGTTCAAGTACTCGCACCGCCGCCACCACTCCAACACCAACTCCGTCGAGCACGACGAGGTCTTCGTCCCCCGCCACAAGGACGGCGTCCAGTGGTACTACCGCTTCTTCAACAACACCCCGGGCCGCGTCCTCACCCTCACGCTCACGCTCCTCGTCGGCTGGCCGTCGTACCTCGCGTTCAACGCGTCGGGCCGCCCGTACGACGGCTTCGCGTCCCACTACAACCCGAACGCGCAGATCTTCAACCTCCGCGAGCGCTTCTGGGTCCACGTCTCGAACATCGGCATCCTCGCGATCTACTACATCCTCTACCGCCTCGCGACGACCAAGGGCCTCCCGTGGCTCCTCTCGATCTACGGCGTCCCGGTCCTCATCCTCAACGCCTTCGTCGTCCTCATCACGTTCCTCCAGCACTCGCACCCGGCGCTCCCGCACTACAACTCGGACGAGTGGGACTGGCTCCGCGGCGCGCTCGCGACGGTCGACCGCGACTACGGCTTCCTCAACGAGGTCTTCCACGACATCACGGACACGCACGTCATCCACCACCTCTTCCCGACCATGCCCCACTACAACGCCAAGGAGGCGACCGTCTCGATCCGCCCGATCCTCAAGGACTACTACAAGTTCGACCGCACCCCGATCTGGCGCGCGCTCTGGCGCGAGGCCAAGGAGTGCCTCTACGTCGAGGCGGACGGCACGGGCTCCAAGGGCGTCCTCTGGTTCAAGTCGAAGTTCTAG
**T***PMA1* (PQ281408)	gcctgtgcttcgcctagactgcgattcggctccagttcctttcgcctttctcccgcttttccctttcatcgttagtcccctccatccccgtcgtctcccccctcgctctcgccttcgcatattcccccctcacatccgctcgtctctccgcgtgctctctctctcgctctctcgtcgttaatatcagactcacacaagttcctagagtacacaaagactggttcttcgcacatcgttcgttcgccgtgagaggaggagaggagaggagagcaagttggtctgtctacccgtcccgccgcttcgcttcgcttcgcttggcctgtcgcttgtctgctcgtgtgcctgctagcacagtactgctgctcgagccatccgttcaccgccgtcgccatcgccgccgccgactgtctgctcctgtcctcgacttccgcaactccgctctttacgcctcatcat

Table S3. Fatty acid composition of wild type strain IFO0880 and *PgFADX*-expressing strains cultivated in MedA^{+} medium containing 6% glucose for 72 h, 120 h, and 168 h.

**72 h**	**IFO**	**PGI26**	**PGI28**	**NAR13**	**NAR16**	**PMA5**	**PMA6**
**C16:0**	20.31 ± 0.28	22.07 ± 0.64	21.27 ± 0.39	20.10 ± 0.08	20.04 ± 0.64	21.47 ± 0.31	20.08 ± 0.12
**C16:1**	0.26 ± 0.01	0.30 ± 0.02	0.28 ± 0.01	0.25 ± 0.01	0.24 ± 0.01	0.39 ± 0.04	0.35 ± 0.01
**C18:0**	13.30 ± 0.37	11.04 ± 0.50	11.39 ± 0.33	12.50 ± 0.14	12.90 ± 0.37	9.61 ± 0.11	9.60 ± 0.18
**C18:1**	46.28 ± 0.08	50.86 ± 0.04	51.73 ± 0.19	52.50 ± 0.01	52.09 ± 0.16	54.52 ± 0.21	56.41 ± 0.02
**C18:2**	13.70 ± 0.03	11.37 ± 0.04	11.07 ± 0.11	9.87 ± 0.06	9.78 ± 0.05	10.13 ± 0.25	9.29 ± 0.03
**C18:3**	2.97 ± 0.06	1.83 ± 0.07	2.03 ± 0.07	2.41 ± 0.03	2.58 ± 0.04	0.83 ± 0.03	0.66 ± 0.04
**PuA**	-	0.13 ± 0.00	0.10 ± 0.00	0.13 ± 0.00	0.07 ± 0.00	0.97 ± 0.11	1.32 ± 0.06
**C20:0**	0.42 ± 0.01	0.37 ± 0.01	0.37 ± 0.01	0.40 ± 0.01	0.41 ± 0.01	0.34 ± 0.01	0.35 ± 0.01
**C22:0**	0.74 ± 0.02	0.61 ± 0.00	0.57 ± 0.02	0.60 ± 0.01	0.62 ± 0.01	0.54 ± 0.01	0.57 ± 0.01
**C24:0**	2.06 ± 0.05	1.44 ± 0.01	1.21 ± 0.05	1.25 ± 0.04	1.31 ± 0.01	1.22 ± 0.02	1.41 ± 0.04
**SFA**	36.81 ± 0.18	35.53 ± 0.13	34.80 ± 0.02	34.84 ± 0.13	35.27 ± 0.24	33.17 ± 0.22	31.99 ± 0.04
**MUFA**	46.54 ± 0.09	51.16 ± 0.02	52.01 ± 0.18	52.75 ± 0.03	52.33 ± 0.15	54.91 ± 0.16	56.75 ± 0.01
**PUFA**	16.67 ± 0.09	13.33 ± 0.11	13.20 ± 0.18	12.41 ± 0.09	12.43 ± 0.09	11.92 ± 0.38	11.27 ± 0.05
**MUFA/PUFA**	2.79 ± 0.01	3.84 ± 0.03	3.94 ± 0.07	4.25 ± 0.03	4.21 ± 0.02	4.61 ± 0.16	5.04 ± 0.02
**UFA/SFA**	1.72 ± 0.01	1.82 ± 0.01	1.87 ± 0.00	1.87 ± 0.01	1.84 ± 0.02	2.01 ± 0.02	2.13 ± 0.00

**120 h**	**IFO**	**PGI26**	**PGI28**	**NAR13**	**NAR16**	**PMA5**	**PMA6**
**C16:0**	20.16 ± 0.06	21.68 ± 0.74	20.79 ± 0.30	19.81 ± 0.04	19.76 ± 0.56	21.18 ± 0.14	19.89 ± 0.15
**C16:1**	0.27 ± 0.00	0.30 ± 0.03	0.28 ± 0.01	0.26 ± 0.01	0.24 ± 0.01	0.35 ± 0.01	0.35 ± 0.01
**C18:0**	12.83 ± 0.26	10.74 ± 0.40	11.12 ± 0.27	11.88 ± 0.07	12.34 ± 0.32	9.36 ± 0.11	9.31 ± 0.16
**C18:1**	46.63 ± 0.03	51.03 ± 0.21	52.18 ± 0.07	52.87 ± 0.00	52.50 ± 0.22	54.62 ± 0.16	56.33 ± 0.15
**C18:2**	14.01 ± 0.17	11.85 ± 0.09	11.34 ± 0.07	10.46 ± 0.01	10.26 ± 0.01	10.62 ± 0.19	9.94 ± 0.11
**C18:3**	3.25 ± 0.13	1.99 ± 0.06	2.02 ± 0.04	2.42 ± 0.01	2.58 ± 0.01	0.95 ± 0.03	0.74 ± 0.00
**PuA**	-	0.12 ± 0.00	0.10 ± 0.00	0.13 ± 0.01	0.07 ± 0.00	0.98 ± 0.01	1.27 ± 0.03
**C20:0**	0.41 ± 0.01	0.35 ± 0.01	0.35 ± 0.01	0.37 ± 0.01	0.38 ± 0.01	0.31 ± 0.00	0.32 ± 0.00

**C22:0**	0.69 ± 0.03	0.58 ± 0.00	0.57 ± 0.01	0.57 ± 0.00	0.60 ± 0.01	0.51 ± 0.01	0.53 ± 0.01
**C24:0**	1.78 ± 0.10	1.38 ± 0.01	1.26 ± 0.06	1.25 ± 0.01	1.30 ± 0.00	1.14 ± 0.00	1.34 ± 0.03
**SFA**	35.86 ± 0.33	34.72 ± 0.32	34.09 ± 0.05	33.87 ± 0.04	34.36 ± 0.23	32.50 ± 0.04	31.39 ± 0.01
**MUFA**	46.90 ± 0.03	51.33 ± 0.18	52.46 ± 0.06	53.13 ± 0.01	52.73 ± 0.21	54.97 ± 0.16	56.68 ± 0.13
**PUFA**	17.26 ± 0.30	13.96 ± 0.15	13.46 ± 0.11	13.01 ± 0.01	12.91 ± 0.02	12.55 ± 0.21	11.95 ± 0.13
**MUFA/PUFA**	2.72 ± 0.05	3.68 ± 0.03	3.90 ± 0.04	4.08 ± 0.00	4.09 ± 0.01	4.38 ± 0.08	4.75 ± 0.06
**UFA/SFA**	1.79 ± 0.03	1.88 ± 0.03	1.93 ± 0.00	1.95 ± 0.00	1.91 ± 0.02	2.08 ± 0.00	2.19 ± 0.00

**168 h**	**IFO**	**PGI26**	**PGI28**	**NAR13**	**NAR16**	**PMA5**	**PMA6**
**C16:0**	19.77 ± 0.01	21.50 ± 0.98	20.63 ± 0.37	19.57 ± 0.12	19.60 ± 0.54	20.94 ± 0.25	19.72 ± 0.06
**C16:1**	0.27 ± 0.01	0.30 ± 0.03	0.28 ± 0.01	0.25 ± 0.00	0.24 ± 0.01	0.35 ± 0.01	0.35 ± 0.01
**C18:0**	12.15 ± 0.22	10.24 ± 0.54	10.71 ± 0.32	11.58 ± 0.03	12.00 ± 0.16	8.90 ± 0.45	9.00 ± 0.08
**C18:1**	46.94 ± 0.11	51.31 ± 0.45	52.43 ± 0.17	53.10 ± 0.03	52.72 ± 0.20	54.95 ± 0.05	56.64 ± 0.13
**C18:2**	14.77 ± 0.14	12.35 ± 0.11	11.72 ± 0.14	10.79 ± 0.16	10.58 ± 0.18	11.10 ± 0.28	10.24 ± 0.04
**C18:3**	3.28 ± 0.08	2.04 ± 0.00	2.06 ± 0.06	2.43 ± 0.05	2.63 ± 0.08	0.97 ± 0.03	0.76 ± 0.01
**PuA**	-	0.12 ± 0.00	0.09 ± 0.00	0.13 ± 0.00	0.07 ± 0.00	0.96 ± 0.01	1.25 ± 0.02
**C20:0**	0.37 ± 0.01	0.31 ± 0.01	0.33 ± 0.01	0.35 ± 0.00	0.36 ± 0.01	0.29 ± 0.02	0.30 ± 0.01
**C22:0**	0.67 ± 0.02	0.54 ± 0.03	0.54 ± 0.03	0.56 ± 0.01	0.57 ± 0.01	0.48 ± 0.04	0.50 ± 0.01
**C24:0**	1.81 ± 0.07	1.31 ± 0.09	1.22 ± 0.07	1.24 ± 0.02	1.26 ± 0.05	1.09 ± 0.13	1.28 ± 0.07
**SFA**	34.75 ± 0.33	33.89 ± 0.30	33.43 ± 0.06	33.29 ± 0.18	33.78 ± 0.45	31.68 ± 0.38	30.79 ± 0.08
**MUFA**	47.21 ± 0.12	51.61 ± 0.42	52.71 ± 0.16	53.35 ± 0.03	52.96 ± 0.19	55.30 ± 0.06	56.99 ± 0.12
**PUFA**	18.05 ± 0.23	14.51 ± 0.11	13.87 ± 0.21	13.35 ± 0.21	13.27 ± 0.25	13.03 ± 0.32	12.24 ± 0.05
**MUFA/PUFA**	2.62 ± 0.03	3.56 ± 0.06	3.80 ± 0.07	4.00 ± 0.06	3.99 ± 0.06	4.25 ± 0.10	4.66 ± 0.03
**UFA/SFA**	1.88 ± 0.03	1.95 ± 0.03	1.99 ± 0.01	2.00 ± 0.02	1.96 ± 0.04	2.16 ± 0.04	2.25 ± 0.01

Abbreviations: MUFA, monounsaturated fatty acids; PuA, punicic acid; PUFA, polyunsaturated fatty acids; SFA, saturated fatty acids; UFA, unsaturated fatty acids.

Table S4. Fatty acid composition of wild type strain IFO0880 and *PgFADX*-expressing strains cultivated in MedA^{+} medium containing 6% crude glycerol for 72 h, 120 h, and 168 h.

**72 h**	**IFO**	**PGI26**	**PGI28**	**NAR13**	**NAR16**	**PMA5**	**PMA6**
**C16:0**	17.65 ± 0.50	19.11 ± 0.47	18.67 ± 0.67	18.25 ± 0.62	17.67 ± 0.01	20.07 ± 0.63	18.02 ± 0.11
**C16:1**	0.34 ± 0.01	0.40 ± 0.02	0.37 ± 0.01	0.40 ± 0.02	0.37 ± 0.00	0.53 ± 0.02	0.45 ± 0.01
**C18:0**	17.84 ± 0.54	16.45 ± 0.62	17.11 ± 0.39	16.66 ± 0.42	17.01 ± 0.00	13.67 ± 0.42	15.22 ± 0.15
**C18:1**	47.07 ± 0.06	47.89 ± 0.33	46.88 ± 0.25	45.82 ± 0.04	46.22 ± 0.29	51.79 ± 0.22	51.61 ± 0.11
**C18:2**	11.98 ± 0.01	11.50 ± 0.09	12.09 ± 0.06	13.36 ± 0.14	13.27 ± 0.21	10.18 ± 0.05	10.47 ± 0.03
**C18:3**	2.01 ± 0.02	1.69 ± 0.06	1.90 ± 0.05	2.12 ± 0.04	2.10 ± 0.02	0.74 ± 0.01	0.80 ± 0.01
**PuA**	-	0.04 ± 0.00	0.04 ± 0.01	0.14 ± 0.01	0.09 ± 0.01	0.89 ± 0.08	0.75 ± 0.00
**C20:0**	0.54 ± 0.01	0.50 ± 0.02	0.51 ± 0.02	0.50 ± 0.01	0.51 ± 0.00	0.41 ± 0.01	0.48 ± 0.01
**C22:0**	0.81 ± 0.02	0.75 ± 0.01	0.77 ± 0.01	0.80 ± 0.02	0.81 ± 0.01	0.61 ± 0.01	0.71 ± 0.01
**C24:0**	1.78 ± 0.01	1.68 ± 0.02	1.69 ± 0.01	1.99 ± 0.04	1.98 ± 0.04	1.14 ± 0.00	1.54 ± 0.02
**SFA**	38.61 ± 0.08	38.48 ± 0.21	38.73 ± 0.24	38.19 ± 0.12	37.97 ± 0.06	35.90 ± 0.18	35.95 ± 0.08
**MUFA**	47.41 ± 0.07	48.29 ± 0.35	47.25 ± 0.24	46.21 ± 0.06	46.59 ± 0.29	52.31 ± 0.20	52.05 ± 0.11
**PUFA**	13.99 ± 0.01	13.22 ± 0.16	14.02 ± 0.01	15.61 ± 0.17	15.45 ± 0.23	11.80 ± 0.03	12.02 ± 0.04
**MUFA/PUFA**	3.39 ± 0.00	3.65 ± 0.07	3.37 ± 0.02	2.96 ± 0.04	3.02 ± 0.06	4.43 ± 0.03	4.33 ± 0.02
**UFA/SFA**	1.59 ± 0.01	1.60 ± 0.01	1.58 ± 0.02	1.62 ± 0.01	1.63 ± 0.00	1.79 ± 0.01	1.78 ± 0.01

**120 h**	**IFO**	**PGI26**	**PGI28**	**NAR13**	**NAR16**	**PMA5**	**PMA6**
**C16:0**	17.31 ± 0.31	18.86 ± 0.27	18.11 ± 0.37	19.00 ± 1.12	18.24 ± 0.41	19.73 ± 0.64	17.83 ± 0.24
**C16:1**	0.35 ± 0.01	0.41 ± 0.01	0.38 ± 0.01	0.41 ± 0.04	0.39 ± 0.01	0.53 ± 0.04	0.45 ± 0.01
**C18:0**	17.46 ± 0.21	15.85 ± 0.33	16.78 ± 0.10	16.12 ± 0.78	16.55 ± 0.42	13.63 ± 0.47	14.94 ± 0.23
**C18:1**	49.13 ± 0.00	49.89 ± 0.34	49.42 ± 0.23	47.90 ± 0.28	48.57 ± 0.12	52.79 ± 0.16	52.80 ± 0.06
**C18:2**	11.07 ± 0.08	10.66 ± 0.17	10.89 ± 0.13	11.72 ± 0.06	11.55 ± 0.09	9.73 ± 0.06	9.95 ± 0.04
**C18:3**	1.92 ± 0.01	1.67 ± 0.07	1.76 ± 0.02	1.96 ± 0.01	1.84 ± 0.02	0.87 ± 0.02	0.90 ± 0.01
**PuA**	-	0.03 ± 0.00	0.03 ± 0.01	0.14 ± 0.01	0.13 ± 0.01	0.69 ± 0.06	0.60 ± 0.00
**C20:0**	0.56 ± 0.01	0.51 ± 0.01	0.55 ± 0.01	0.49 ± 0.02	0.51 ± 0.01	0.44 ± 0.02	0.50 ± 0.01

**C22:0**	0.78 ± 0.01	0.73 ± 0.01	0.75 ± 0.01	0.73 ± 0.01	0.74 ± 0.01	0.62 ± 0.01	0.71 ± 0.01
**C24:0**	1.45 ± 0.01	1.39 ± 0.04	1.37 ± 0.04	1.53 ± 0.01	1.51 ± 0.01	1.01 ± 0.01	1.34 ± 0.02
**SFA**	37.56 ± 0.06	37.32 ± 0.11	37.54 ± 0.33	37.86 ± 0.31	37.54 ± 0.03	35.41 ± 0.15	35.31 ± 0.03
**MUFA**	49.48 ± 0.01	50.30 ± 0.35	49.80 ± 0.22	48.31 ± 0.24	48.96 ± 0.13	53.31 ± 0.13	53.25 ± 0.07
**PUFA**	12.98 ± 0.07	12.36 ± 0.24	12.67 ± 0.11	13.82 ± 0.06	13.51 ± 0.11	11.28 ± 0.01	11.44 ± 0.04
**MUFA/PUFA**	3.81 ± 0.02	4.07 ± 0.11	3.93 ± 0.02	3.50 ± 0.00	3.63 ± 0.04	4.73 ± 0.01	4.65 ± 0.02
**UFA/SFA**	1.66 ± 0.00	1.68 ± 0.01	1.66 ± 0.02	1.64 ± 0.02	1.66 ± 0.00	1.82 ± 0.01	1.83 ± 0.00

**168 h**	**IFO**	**PGI26**	**PGI28**	**NAR13**	**NAR16**	**PMA5**	**PMA6**
**C16:0**	17.08 ± 0.32	18.71 ± 0.04	17.86 ± 0.72	18.74 ± 1.12	17.90 ± 0.26	19.45 ± 0.74	17.54 ± 0.10
**C16:1**	0.36 ± 0.01	0.44 ± 0.01	0.39 ± 0.03	0.43 ± 0.04	0.41 ± 0.01	0.54 ± 0.04	0.46 ± 0.00
**C18:0**	16.82 ± 0.28	15.20 ± 0.14	16.20 ± 0.46	15.59 ± 0.86	15.94 ± 0.33	13.28 ± 0.50	14.45 ± 0.01
**C18:1**	49.77 ± 0.01	50.51 ± 0.57	50.25 ± 0.40	49.40 ± 0.21	50.10 ± 0.31	53.32 ± 0.22	53.25 ± 0.03
**C18:2**	11.28 ± 0.00	10.88 ± 0.28	10.93 ± 0.11	11.25 ± 0.02	11.15 ± 0.18	9.82 ± 0.04	10.27 ± 0.11
**C18:3**	2.06 ± 0.01	1.76 ± 0.06	1.82 ± 0.06	1.86 ± 0.04	1.72 ± 0.06	0.93 ± 0.01	0.99 ± 0.01
**PuA**	-	0.03 ± 0.00	0.03 ± 0.01	0.26 ± 0.04	0.29 ± 0.02	0.68 ± 0.06	0.61 ± 0.01
**C20:0**	0.56 ± 0.01	0.50 ± 0.00	0.54 ± 0.03	0.50 ± 0.04	0.52 ± 0.01	0.45 ± 0.02	0.50 ± 0.00
**C22:0**	0.76 ± 0.02	0.71 ± 0.01	0.74 ± 0.02	0.70 ± 0.03	0.71 ± 0.01	0.62 ± 0.02	0.69 ± 0.00
**C24:0**	1.33 ± 0.04	1.29 ± 0.05	1.28 ± 0.01	1.30 ± 0.01	1.29 ± 0.01	0.95 ± 0.01	1.25 ± 0.01
**SFA**	36.54 ± 0.04	36.40 ± 0.23	36.61 ± 0.21	36.82 ± 0.18	36.35 ± 0.11	34.74 ± 0.18	34.43 ± 0.09
**MUFA**	50.13 ± 0.02	50.95 ± 0.57	50.64 ± 0.37	49.83 ± 0.16	50.51 ± 0.33	53.85 ± 0.18	53.71 ± 0.03
**PUFA**	13.34 ± 0.01	12.66 ± 0.34	12.77 ± 0.18	13.36 ± 0.02	13.15 ± 0.21	11.43 ± 0.01	11.86 ± 0.11
**MUFA/PUFA**	3.76 ± 0.00	4.03 ± 0.15	3.97 ± 0.08	3.73 ± 0.01	3.84 ± 0.09	4.71 ± 0.02	4.53 ± 0.05
**UFA/SFA**	1.74 ± 0.00	1.75 ± 0.02	1.73 ± 0.02	1.72 ± 0.01	1.75 ± 0.01	1.88 ± 0.01	1.90 ± 0.01

Abbreviations: MUFA, monounsaturated fatty acids; PuA, punicic acid; PUFA, polyunsaturated fatty acids; SFA, saturated fatty acids; UFA, unsaturated fatty acids.
